# Supplementary material for: Class attendance, peer similarity, and academic performance in a large field study
Source: PLoS One. 2017 Nov 8;12(11):e0187078. doi: 10.1371/journal.pone.0187078 (PMC5678706; doi:10.1371/journal.pone.0187078)
Supplement: S1 File — The supporting information contains further details on the data collection process and the recorded data types. Additionally, we discuss our method on correcting attendance data on the class level and show that we observe distinct attendance-performance correlations for courses of different subject areas. (PDF) [file pone.0187078.s001.pdf]

Dataset details

Data collection

During the Copenhagen Network Study, we collected data in various channels about 1,000 students for a period of 2 years [35]. Collection of the data was carried out by the use of dedicated Android smart phones with an open source based application running in the background. Our data collector application was built on top of existing technologies such as the FUNF project (developed by the Media Lab, MIT) as described in details in [35]. To avoid data loss, we flashed the phones, that is, we extended the operating system of the phones to include our data collector. The phones were handed out to students free of charge for participating in the experiment voluntarily.

An important aspect of the experiment is the way participants were engaged in the data collection and the complete transparency towards them. First, students could exit the experiment at any time. Second, the data collected by the phone was available for the participants, and an interactive webpage was provided where students had the opportunity to see their own data as well as to enable or disable any of the data channels. In the early months of the experiment, the collector was accompanied by a summary application that showed basic statistics to the students from the data we obtained [35].

The data we have recorded include call detail records (CDR) together with meta data on text messages, Facebook activity data (using the Graph API), location (using GPS and WiFi router scans), proximity links using Bluetooth scans and screen on/off events. Temporal resolution varies over the data types, but for each channel we have a time window of at most 5 minutes. Prior to the experiment, students participated in a psychological survey which provides us with the standard psychological traits and metrics. This high dimensionality of data sources allows us to compare channels and to assess more complex behavioral patterns. Measuring proximity between students enables us to construct physical proximity networks that are essential for estimating class location and class attendance with a higher accuracy than, e.g., comparing GPS locations.

Finally, the day and time of each course is obtained at kurser.dtu.dk, which is a database of all applicable courses, in yearly breakdown. Grade data is provided by the Technical University of Denmark, and includes all final grades, that is, any valid grade that is the result of an exam, project or absence from the exam.

Correcting for course-level attendance

Attendance measured in various classes depends on different factors. One possible explanation for the observed correlations is the individual aspects of the courses: some are entertaining and involving, thus students were highly motivated to attend the classes, while others required less activity and therefore the attendance dropped. To account for these course level differences, we calculate the corrected attendance for each class, that is, the attendance of the students relative to the average attendance in the class. This way, individual differences at the level of courses (and classes) are eliminated and the resulting attendance measures are independent of the actual course. To assess the strength of peer-similarity, we plot the average attendance of the strongest contacts (i.e. communicating by text messages) assigned to the same course for each student as the function of own attendance, shown in Fig 7. The overall structure of the

distribution resembles a straight line, with most of the data points gathering around the diagonal from $(-1,-1)$ to $(1,1)$. The concentration of points around this diagonal suggests a correlation between self and peer behavior with respect to the corrected attendance as well, further supporting the findings in the main paper.

Subject-specific behavior

Correlations vary over the courses, with some courses exhibiting high correlation between attendance and the final grade, and others displaying low or moderate correlations. In Fig A we show the probability density of correlations measured over all courses considered in this paper, as well as density functions restricted to specific areas. As the area plots indicate, the dispersion in the distribution of correlations suggest distinct behavior by field. Courses assigned to mathematics (with lecturers being affiliated to *applied mathematics and computer science* or *informatics and mathematical modeling*) show the lowest correlation values with a median correlation of .126. On the contrary, courses affiliated with *chemistry* and *chemical engineering* are characterized by high correlations, marked by a median value of .427. Note that there are a few courses with negative correlation: these are the artifact of low sample sizes (the corresponding significance is low).

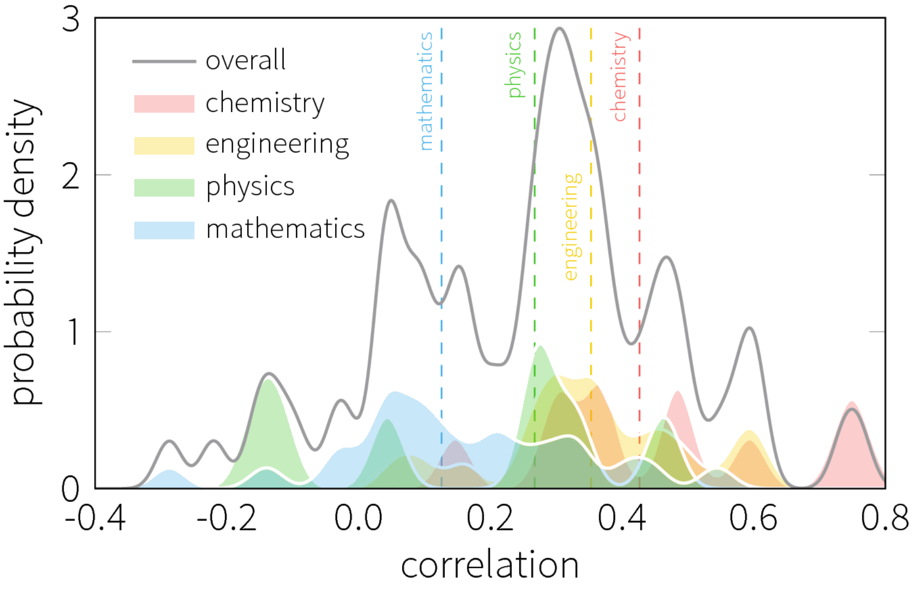

Fig A. Distribution of correlations between attendance and performance. The grey curve shows the overall probability density of Spearman correlations measured over all courses. Area plots denote the probability density measured for specific fields, dashed lines correspond to the median correlation observed in the field. Scale of the field density plots is shrinked and used only for illustrative purposes.
